# Functional role of cyanidin-3-O-glucoside in osteogenesis: A pilot study based on RNA-seq analysis

**DOI:** 10.3389/fnut.2022.995643

**Published:** 2022-09-30

**Authors:** Lin Chen, Bosen Hu, Xiaohong Wang, Yong Chen, Bo Zhou

**Affiliations:** ^1^School of Public Health, Shenyang Medical College, Shenyang, China; ^2^Central Hospital Affiliated to Shenyang Medical College, Shenyang, China

**Keywords:** cyanidin-3-O-glucoside, osteogenesis, RNA-sequencing, MC3T3-E1 cells, *Atp6v0c*, *Cx3cl1*, *Ly6a*

## Abstract

Cyanidin-3-O-glucoside (C3G) is the most widely distributed anthocyanin and it can reportedly reduce the risk of osteoporosis, but the molecular mechanism by which C3G promotes bone formation is poorly understood. In the current study, RNA sequencing (RNA-seq) was used to investigate the mechanism of action of C3G in osteogenesis. MC3T3-E1 mouse osteoblasts were divided into a C3G (100 μmol/L)-treated group and a vehicle-treated control group, and differentially expressed genes (DEGs) in groups were evaluated *via* RNA-seq analysis. The functions of the DEGs were evaluated by Gene Ontology (GO) and Kyoto Encyclopedia of Genes and Genomes (KEGG) analyses, and the genes were validated by quantitative real-time PCR. The RNA-seq analysis identified 34 genes that were upregulated in C3G-treated cells compared to vehicle-treated cells, and 17 that were downregulated GO and KEGG pathway analyses indicated that these genes were highly enriched in functions related to lysosomes and glycolipid biosynthesis, among others. The differential expression of ATPase H+-transporting V0 subunit C (*Atp6v0c*), chemokine (C-X3-C motif) ligand 1 (*Cx3cl1*), and lymphocyte antigen 6 complex, locus A (*Ly6a*) genes was validated by quantitative real-time-PCR. Because these genes have been previously implicated in osteoporosis, they are potential target genes of C3G action in MC3T3-E1 cells. These results provide molecular level evidence for the therapeutic potential of C3G in the treatment of osteoporosis and other disorders of bone metabolism.

## Introduction

Osteoporosis is a disorder of bone metabolism characterized by reduced bone mineral density and a high risk of bone fracture (1). It is a global public health problem (2) that will worsen with the increasing life expectancy of the human population. Osteoblasts and osteoclasts are responsible for bone remodeling, which maintains the integrity of the skeleton (3, 4). Primary cause of osteoporosis is dysfunctional osteoblasts and osteoclasts activity (5). Promoting the proliferation and differentiation of osteoblasts is an effective way to enhance bone mineral density and prevent osteoporosis (6).

Cyanidin-3-O-glucoside (C3G) is an anthocyanin that is widely distributed in nature (7, 8). C3G evidently has antioxidant and anti-inflammatory effects, therapeutic effects on disease, such as obesity, type 2 diabetes mellitus, and prostate cancer (9, 10), and promotes bone formation and reduces bone loss (11–16). In a study conducted in the United Kingdom, women with high dietary anthocyanin intake had higher bone mineral density (11, 12). In rodents, C3G supplementation can reportedly improve bone quality and reduce bone loss (13, 17, 18), and in other studies it has enhanced osteoblast differentiation and mineralization (19, 20). C3G may be a natural product supporting the prevention and treatment of osteoporosis (21).

To date most studies investigating the molecular etiology of osteoporosis have focused on components of signaling pathways related to bone development such as mitogen-activated protein kinase (MAPK) (22, 23), nuclear factor kappa B (NF-κB) (22), bone morphogenetic protein (BMP) (24), and Wnt (25) pathways. Recent studies have also revealed roles for the chemokine (C-X3-C motif) ligand 1 (CX3CL1)/chemokine (C-X3-C motif) receptor 1 (CX3CR1) signaling axis (26) and glycosylphosphatidylinositol-anchored proteins (27).

Anthocyanins from black rice, which is enriched in C3G (28) and blackberry (29) were shown to affect osteoblast proliferation and differentiation by modulating the expression of target genes including alkaline phosphatase (*Alp*), osteopontin (*Opn*), osterix (*Osx*), and bone gamma-carboxyglutamic acid-containing protein (*Bglap*) (20). C3G increased the mineralization capacity of osteoblasts via the extracellular signal regulated kinase 1/2 (ERK1/2) signaling pathway (19). Several molecular mechanisms of C3G have been investigated in bone cells, but its effects on gene regulation involved in bone formation remain largely unknown.

RNA sequencing (RNA-seq) technology combined with bioinformatics have enabled the large-scale identification of genes associated with normal biological processes and pathogenic processes (30). In current study, gene expression profiles of ME3T3-E1 osteoblast-like cells with and without C3G treatment were investigated by RNA-seq and a functional analyses of differentially expressed genes (DEGs) was conducted to identify those that potentially mediate the protective effects of C3G in osteoporosis.

## Materials and methods

### Chemicals and reagents

C3G with the purity over 98% was purchased from Meilunbio (Dalian, China). TRIzol reagent, primers of quantitative polymerase chain reaction (*qRT-PCR*), a reverse transcription kit and SYBR Green MasterMix were purchased from Takara Bio (Ostu, Japan). Pancreatin were purchased from Hyclone (Logan, UT, USA). Dimethylsulfoxide (DMSO), phosphate buffered saline (PBS), and other chemicals were purchased from Sigma-Aldrich (Sigma, USA).

### Cell culture

Murine preosteoblast MC3T3-E1 cells obtained from the Cell Bank of the Chinese Academy of Science (Shanghai, China) were cultured in alpha-minimal essential medium supplemented with 10% fetal bovine serum and 1% penicillin/streptomycin (all from Hyclone, Logan, UT, USA) at 37°C and 5% CO_2_. In a previous study, 100 μmol/L C3G promoted cell proliferation of MC3T3-E1 cells (19), therefore, 7^*^10^5^/ml MC3T3-E1 cells were seeded in 6-well plates in the present study. After the cells had adhere, the cells were synchronized for 24 h using serum-free medium. After completion of the synchronization treatment, the serum-free medium was replaced with complete medium with or without 100 μmol/L C3G, and the cells were culture for a further 24 h.

### RNA-seq analysis

Total RNA was extracted from cells using TRIzol reagent and RNA integrity was evaluated *via* agarose gel electrophoresis and spectrophotometry using a NanoDrop ND-1000 instrument (Thermo Fisher Scientific, Waltham, MA, USA). An RNA library was constructed with the KAPA Stranded RNA-seq Library Prep Kit (Illumina, San Diego, CA, USA), and the quality of the library was assessed using 2100 Bioanalyzer (Agilent Technologies, Santa Clara, CA, USA). Quantification of the library was performed *via* quantitative real-time PCR (qRT-PCR). Sequencing was performed over 150 cycles using the Xten/NovaSeq system (Illumina). Raw RNA-seq data were submitted to NCBI Gene Expression Omnibus (accession number. GSE149731).

### Functional analysis of identified genes

FastQC v0.11.8 was used to analyze the raw RNA-seq data (http://www.bioinformatics.babraham.ac.uk/projects/fastqc/) (31). Fragments per kilobase of gene/transcript model per million mapped fragments values for gene and transcript levels were calculated with the Ballgown package (https://www.bioconductor.org/packages/release/bioc/html/ballgown.html) of R v2.10.0 software. R was used to generate volcano plots and heatmaps to further analyze gene expression profiles.

Gene Ontology (GO) functional enrichment analysis of DEGs (www.geneontology.org/) was performed, and genes involved in biological process, cellular component, and molecular function GO categories were identified (32, 33). Kyoto Encyclopedia of Genes and Genomes (KEGG) pathway analysis (www.genome.jp/kegg/) was conducted to identify signaling pathways associated with the DEGs. All pathways were based on the KEGG database (33).

### Quantitative real-time PCR

RNA was extracted from C3G-treated and untreated MC3T3-E1 cells (*n* = 3 replicates each) using TRIzol reagent. cDNA was synthesized using a reverse transcription kit, and SYBR Green MasterMix was used for qRT-PCR in a reaction volume of 20 μL. qRT-PCR was performed on a real-time PCR machine (ABI 7500, Applied Biosystem, Foster, California, USA). The primer sequences used are shown in Table 1. The glyceraldehyde 3-phosphate dehydrogenase (*Gapdh*) gene was used as the internal control to calculate target gene expression levels *via* the cycle threshold (2^−Δ*ΔCt*^) method.

**Table 1:** Primer sequences used in the study.

**Gene**	**Sequence (5^′^-3^′^)**	**Gene**	**Sequence (5^′^-3^′^)**
LSM12-f	CAGCGTTCACAAGCCCAACAAC	Cx3cl1-f	CTACTAGGAGCTGCGACACG
LSM12-r	CACTGAAGCCACCACCACCATC	Cx3cl1-r	TGTCGTCTCCAGGACAATGG
Foxp1-f	CAAGCTGTGCACCCCATACA	Adprhl2-f	TGAGCCGAGAGGAAGTGGTGTC
Foxp1-r	TGTACAAGAAACGGAGGGCG	Adprhl2-r	GCAGCGCAGGAAGCAGTAGATG
Ly6a-f	CCTGCTGGGTAGGTAGGTGCTC	Txndc5-f	GCCGCTGCTCGTAACTCTGTG
Ly6a-r	CCTCTTCACTGTGCTGGCTGTG	Txndc5-r	CCGCTCGTGGGAGGTAGGTG
Cenpx-f	CGGAAGGAACTGGTGAGCAGAC	Ppp1r15a-f	AGCATGGGCACGCCTTAGAAAC
Cenpx-r	ACGGACAGCAGCCTCTAGTACG	Ppp1r15a-r	CCGCCTCCCTCCCAAGTACAG
Defb25-f	ATGCACCTGTGTCCGGATG	Tmem55b-f	CGTACGGAGCCGGTAAACA
Defb25-r	ATGGCATCAACTCTAGAGCAA	Tmem55b-r	TCTTGATGGGAGTGGCTTCG
Pigc-f	AGTAGTCCCCTTCCAAGCCG	Camk2g-f	CCGCCCGAGATCATCAGAAA
Pigc-r	GCTAAATTCCTGCACCAAGCTC	Camk2g-r	CTTGACACCGCCATCTGACT
Atp6v0c-f	ACGAACAGCCTGACACATGCAC	Gm20521-f	CTCTAGCCGGGAGGATGAAAG
Atp6v0c-r	GCCTGGGTGGGAGATGAGTGG	Gm20521-r	CCAACGTAGATAGAGCGGGC
Ccdc115-f	GGTGGAGGAGGGTTGGCTCTC	Nkiras2-F	CGGGAGCAGGTGCGTTTCTATG
Ccdc115-r	GCACGCACGCAGACCTGAG	Nkiras2-R	ACGTAGCCATCGGTGCAGGAG
Ugt1a7c-f	TTGCCTTAGGCTGCACTTCT	Tex2-f	GAGTGGTTCAGGCGGTTCATCC
Ugt1a7c-r	TCCGGAACAACCACTACGAC	Tex2-r	GCTGCTGCTGCGGCTGTG
Nfya-f	CAGCCGTTAATGGTGCAAGT	Iqcd-f	GCGAGAAGCAGGACGAATAC
Nfya-r	GAGGCACCAACTGTATCTGCT	Iqcd-f	CCACCCGCTTCTTGGAATTG
GAPDH-f	TTGTCTCCTGCGACTTCAACA		
GAPDH-r	GTGGTCCCAGGGTTTCTTACTCC		

### Statistical analysis

Data are presented as mean ± standard deviation. Statistical analyses were performed using SPSS v22.0 software (SPSS Inc, Chicago, IL, USA). Comparisons between two groups were performed with the student's *t*-tests, and *p* < 0.05 was deemed to indicate statistical significance.

## Results

### Identification of DEGs

**Figure 1:**
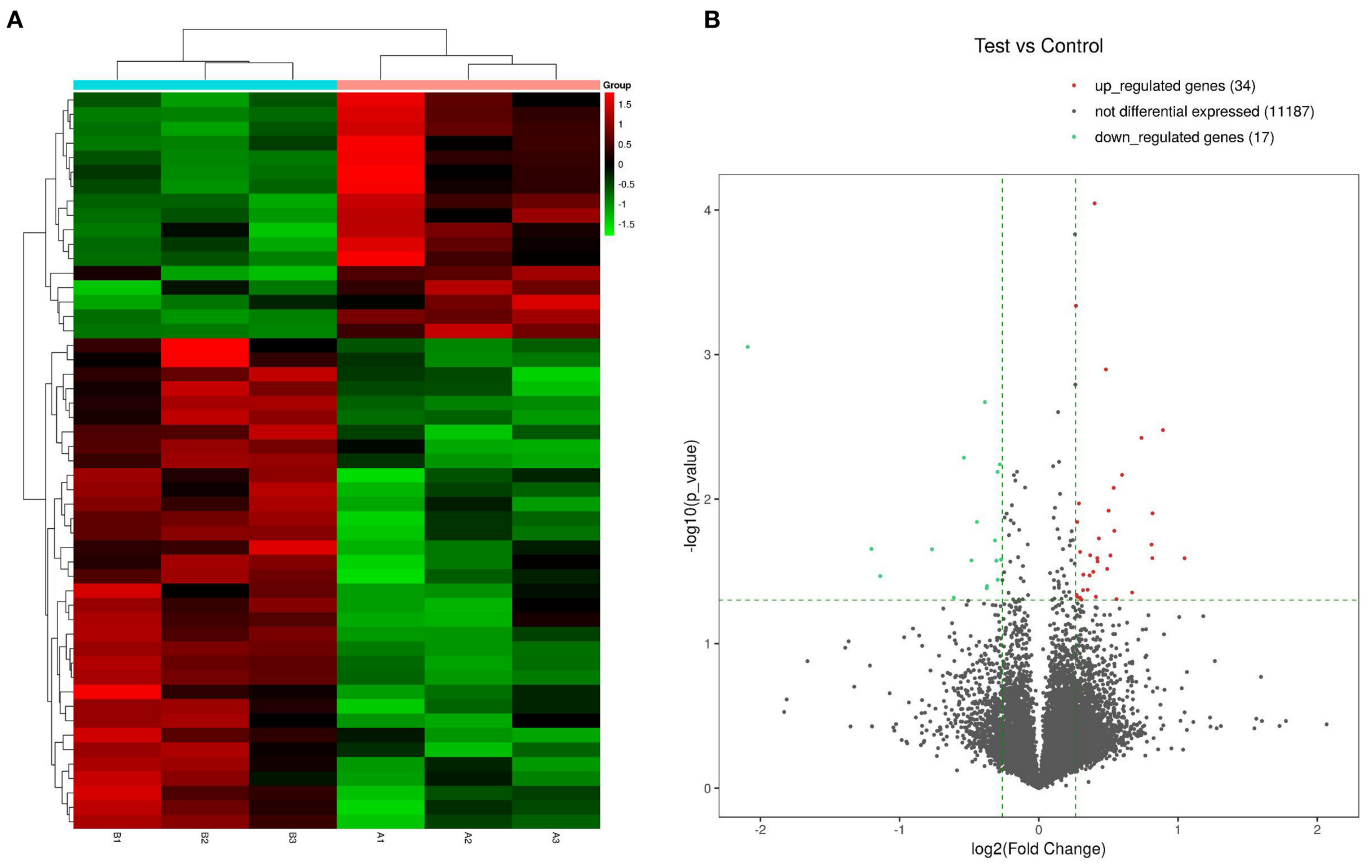
Heatmaps and volcano plot of DEGs in C3G-treated vs. untreated control MC3T3-E1 cells. **(A)** Heatmap of 34 upregulated and 17 downregulated DEGs. **(B)** Volcano plot of upregulated (red dots) and downregulated (green dots) DEGs and genes expressed at a normal level (gray dots).

Expression profiles of 11,238 genes in C3G-treated and untreated MC3T3-E1 cells were determined by RNA-seq, which yielded 51 DEGs (*p* < 0.05, fold change ≥1.2). A heatmap (Figure 1A) and a volcano plot (Figure 1B) were used to represent the abundance of these different transcripts, with expression levels ranging from high (red) to low (green), and there were 34 upregulated and 17 downregulated DEGs. Details of all DEGS are shown in the Supplementary Table 1.

### GO analysis of DEGs

**Figure 2:**
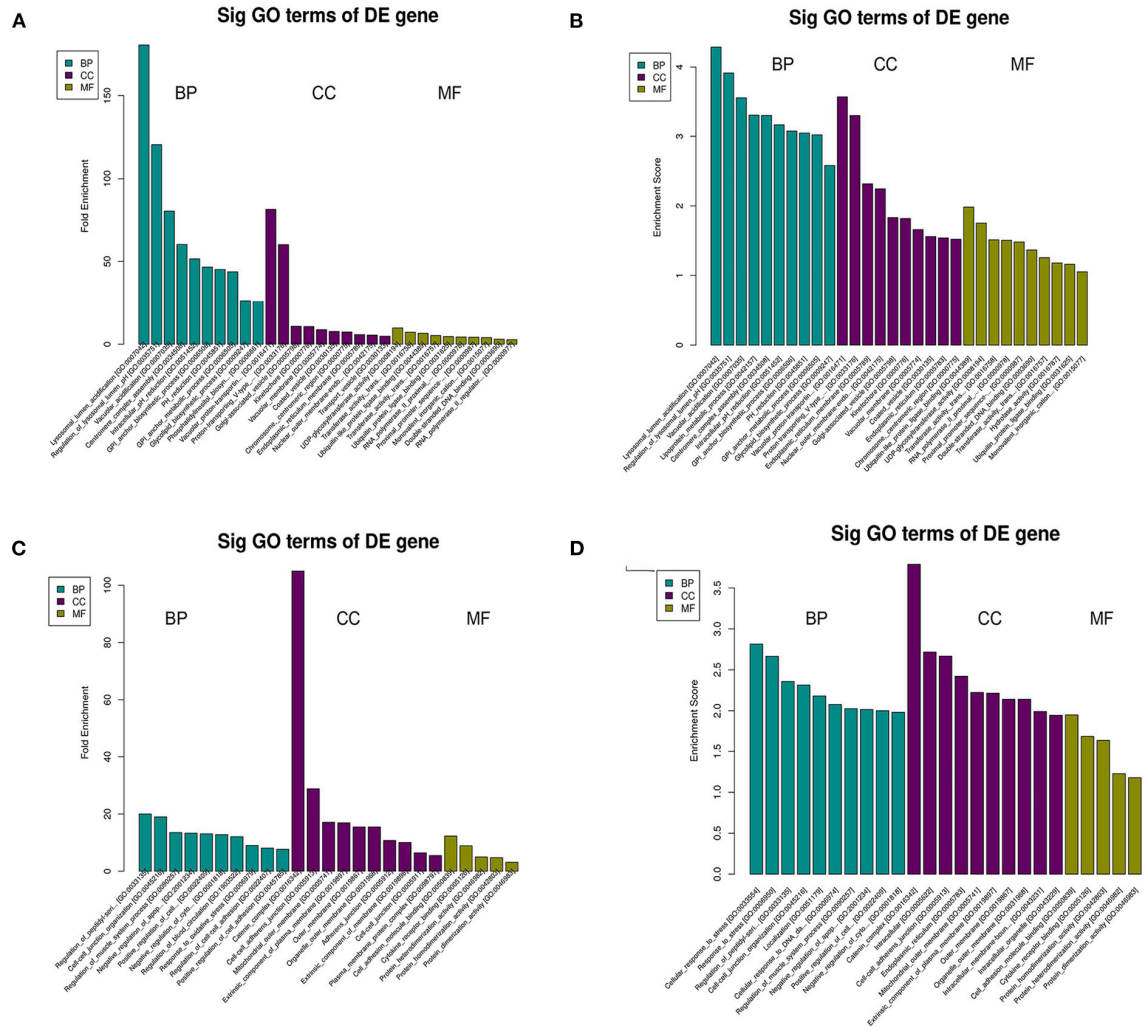
GO analysis of DEGs in C3G-treated MC3T3-E1 cells and untreated control MC3T3-E1 cells. Enriched GO terms corresponding to DEGs in the biological process (BP), cellular component (CC), and molecular function (MF) categories are shown. **(A–D)** GO terms based on upregulated **(A,B)** and downregulated **(C,D)** DEGs.

The top 10 biological process terms from the GO analysis of DEGs are presented in Table 2. All biological process terms are listed in Supplementary Table 2. The top three biological process terms for upregulated DEGs were lysosomal lumen acidification (GO:0007042; *Atp6v0c, Ccdc115*), regulation of lysosomal lumen pH (GO:0035751; *Atp6v0c, Ccdc115*), and lysosome organization (GO:0007040; *Atp6v0c, Ccdc115*). The top three biological process terms for downregulated DEGs were cellular responses to stress (GO:0033554; *Adprhl2, Faap24, Gm20521, Ppp1r15a, Rad1*), response to stress (GO:0006950; *Adprhl2, Camk2g, Cx3cl1, Faap24, Gm20521, Ppp1r15a, Rad1*), and vacuolar acidification (GO:0033135; *Ppp1r15a, Smad7*) for downregulated DEGs. Several significantly enriched entries were related to cellular responses to stress [GO:0033554; ADP-ribosylhydrolase-like 2 (*Adprhl2*), FA core complex-associated protein 24 (*Faap24*), *Gm20521*, protein phosphatase 1 regulatory subunit 15A (*Ppp1r15a*), *Rad1*], cell–cell junction organization [GO:0045216; *Nectin1*, Mothers against decapentaplegic homolog 7 (*Smad7*)], and cellular responses to DNA damage stimuli (GO:0006974; *Faap24, Gm20521, Rad1*). With respect to cellular components (Supplementary Table 3), the most significant terms were ATPase for upregulated DEGs (Figures 2A,B) and outer organelle membranes for downregulated DEGs (Figures 2C,D). The most significant terms pertaining to molecular function (Supplementary Table 4) were ubiquitin-like protein ligase binding for upregulated DEGs (Figures 2A,B) and cell adhesion molecule binding for downregulated DEGs (Figures 2C,D). Other significant GO terms included lipoprotein metabolic process [GO:0042157; lysophospholipase-like 1 (*Lyplal1*), phosphatidylinositol glycan anchor biosynthesis class C (*Pigc*), *Pigk*]; glycosylphosphatidylinositol (GPI) anchor biosynthetic process (GO:0006506; *Pigc, Pigk*); glycolipid biosynthetic process (GO:0009247; *Pigc, Pigk*); GPI-anchor metabolic process (GO:0006505; *Pigc, Pigk*); glycolipid biosynthetic process (GO:0009247; *Pigc, Pigk*); glycolipid metabolic process (GO:0006664; *Pigc, Pigk*); protein lipidation (GO:0006497; *Pigc, Pigk*); and lipoprotein biosynthetic process (GO:0042158; *Pigc, Pigk*).

**Table 2:** Top 10 enriched BP terms of up-regulated and down-regulated DEGs.

**ID**	**Term**	**DEG(s)**	***p*-Value**	**FDR**	**Enrichment**
**Up-regulated**					
GO:0007042	Lysosomal lumen acidification	Atp6v0c, Ccdc115	0.000051713	0.031235009	4.286395308
GO:0035751	Regulation of lysosomal lumen pH	Atp6v0c, Ccdc115	0.000121475	0.036685321	3.915514623
GO:0007035	Vacuolar acidification	Atp6v0c, Ccdc115	0.000280141	0.056401623	3.552624083
GO:0042157	Lipoprotein metabolic process	Lyplal1, Pigc, Pigk	0.000496347	0.060730184	3.30421487
GO:0034508	Centromere complex assembly	Cenpx, Hjurp	0.000502733	0.060730184	3.298662336
GO:0051452	Intracellular pH reduction	Atp6v0c, Ccdc115	0.000686147	0.064043499	3.163582969
GO:0006506	GPI anchor biosynthetic process	Pigc, Pigk	0.000841882	0.064043499	3.074749022
GO:0045851	pH reduction	Atp6v0c, Ccdc115	0.00089723	0.064043499	3.047095975
GO:0006505	GPI anchor metabolic process	Pigc, Pigk	0.000954291	0.064043499	3.020319376
GO:0009247	Glycolipid biosynthetic process	Pigc, Pigk	0.002633397	0.083516259	2.579483631
**Down-regulated**
GO:0033554	Cellular response to stress	Adprhl2, Faap24, Gm20521, Ppp1r15a, Rad1	0.001529696	0.114686788	2.815395
GO:0006950	Response to stress	Adprhl2, Camk2g, Cx3cl1, Faap24, Gm20521, Ppp1r15a, Rad1	0.002156548	0.114686788	2.66624084
GO:0033135	Regulation of peptidyl-serine phosphorylation	Ppp1r15a, Smad7	0.004392513	0.114686788	2.357286896
GO:0045216	cell-cell junction organization	Nectin1, Smad7	0.004863323	0.114686788	2.313066923
GO:0051179	localization	Camk2g, Cx3cl1, Nectin1, Ppp1r15a, Smad7, Tex2, Txndc5, Ubl4a	0.006595983	0.114686788	2.180720442
GO:0006974	Cellular response to DNA damage stimulus	Faap24, Gm20521, Rad1	0.008369015	0.114686788	2.077325642
GO:0090257	Regulation of muscle system process	Camk2g, Smad7	0.009416313	0.114686788	2.026119096
GO:2001234	Negative regulation of apoptotic signaling pathway	Cx3cl1, Gm20521	0.009662976	0.114686788	2.014889099
GO:0022409	Positive regulation of cell-cell adhesion	Cx3cl1, Smad7	0.009996352	0.114686788	2.000158469
GO:0001818	Negative regulation of cytokine production	Cx3cl1, Smad7	0.010420254	0.114686788	1.982121684

### KEGG pathway analysis of DEGs

**Figure 3:**
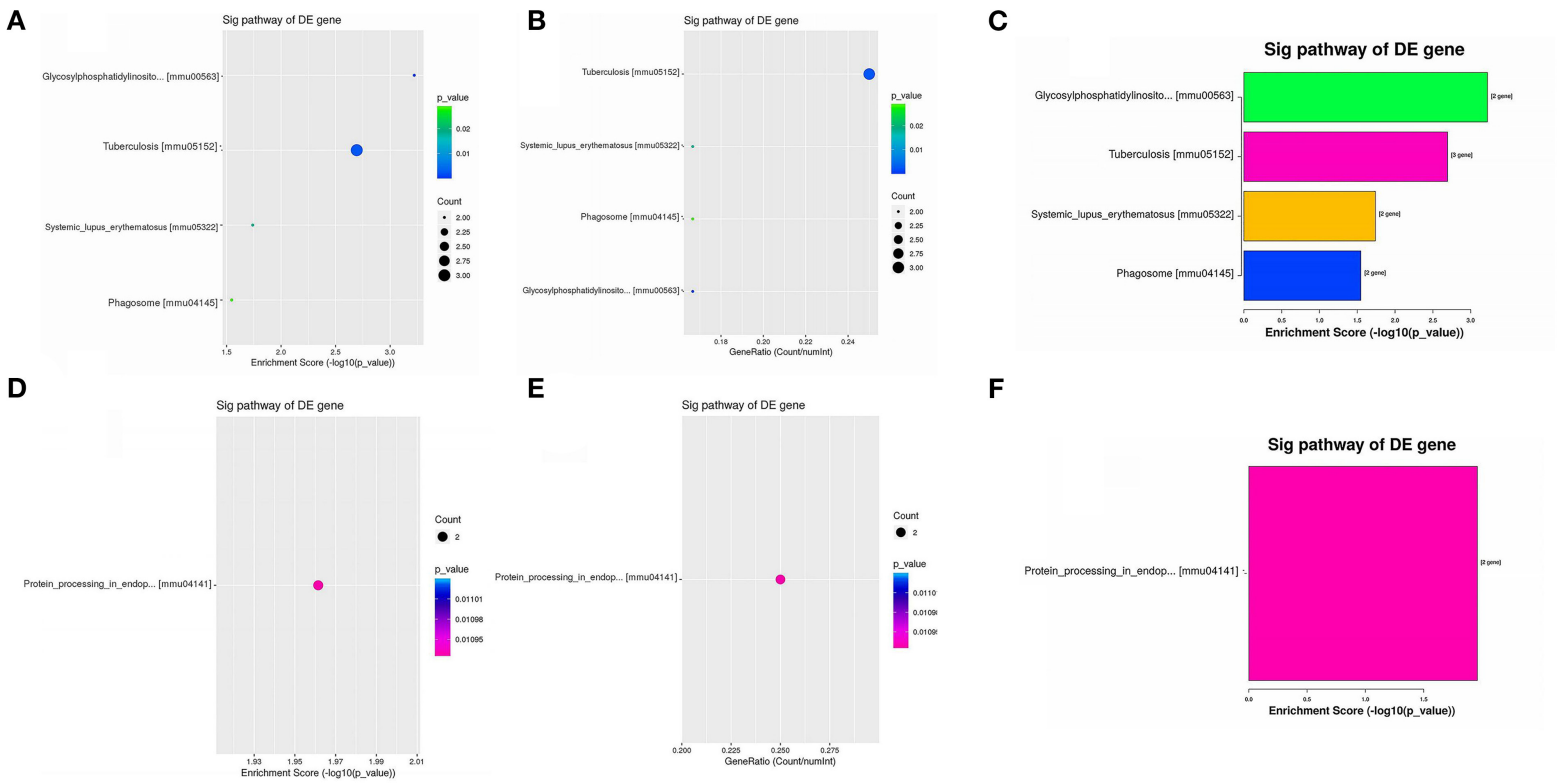
Enriched KEGG pathways of upregulated DEGs and downregulated DEGs. **(A,B)** Dot plots showing the enrichment score (–log10[*p*-value]) of significantly enriched pathways **(A)** and the gene ratio of the top 10 most significantly enriched pathways **(B)**. **(C)** Bar plot showing the top 10 enrichment scores (–log10[*p*-value]) of significantly enriched pathways. **(D,E)** Dot plots showing the enrichment score (–log10[*p*-value]) of the significantly enriched pathways **(D)** and the gene ratios of the top 10 most significantly enriched pathways **(E)**. **(F)** Bar plot showing the top 10 enrichment scores (–log10[*p*-value]) of significantly enriched pathways.

KEGG pathway analysis revealed five significantly enriched signaling pathways (Table 3). Four were upregulated including GPI-anchored protein (GPI-AP) biosynthesis signaling pathway (mmu00563; *Pigc, Pigk*), tuberculosis [mmu05152; *Atp6v0c*, Fc receptor, IgG, low affinity IV (*Fcgr4*), nuclear transcription factor Y subunit alpha (*Nfya*)], Systemic lupus erythematosus [mmu05322; *Fcgr4*, histone H2B (*Hist1h2bq*)], and Phagosome (mmu04145; *Atp6v0c, Fcgr4*) (Figure 3), and one was downregulated protein processing in endoplasmic reticulum [mmu04141; *Ppp1r15a*, thioredoxin domain-containing 5 (*Txndc5*)] (Figure 3).

**Table 3:** Signaling pathway enrichment of up-regulated and down-regulated DEGs.

**ID**	**Term**	**DEG(s)**	***p*-Value**	**FDR**	**Enrichment**
**Up-regulated**
mmu00563	Glycosylphosphatidylinositol (GPI)-anchor biosynthesis	Pigc, Pigk	0.000600808	0.019225871	3.221263961
mmu05152	Tuberculosis	Atp6v0c, Fcgr4, Nfya	0.002024514	0.032392223	2.693679229
mmu05322	Systemic lupus erythematosus	Fcgr4, Hist1h2bq	0.018196375	0.173813623	1.740015125
mmu04145	Phagosome	Atp6v0c, Fcgr4	0.028375513	0.173813623	1.547056275
**Down-regulated**
mmu04141	Protein processing in endoplasmic reticulum	Ppp1r15a, Txndc5	0.000600808	0.199802063	1.961455096

### qRT-PCR analysis of DEGs

**Figure 4:**
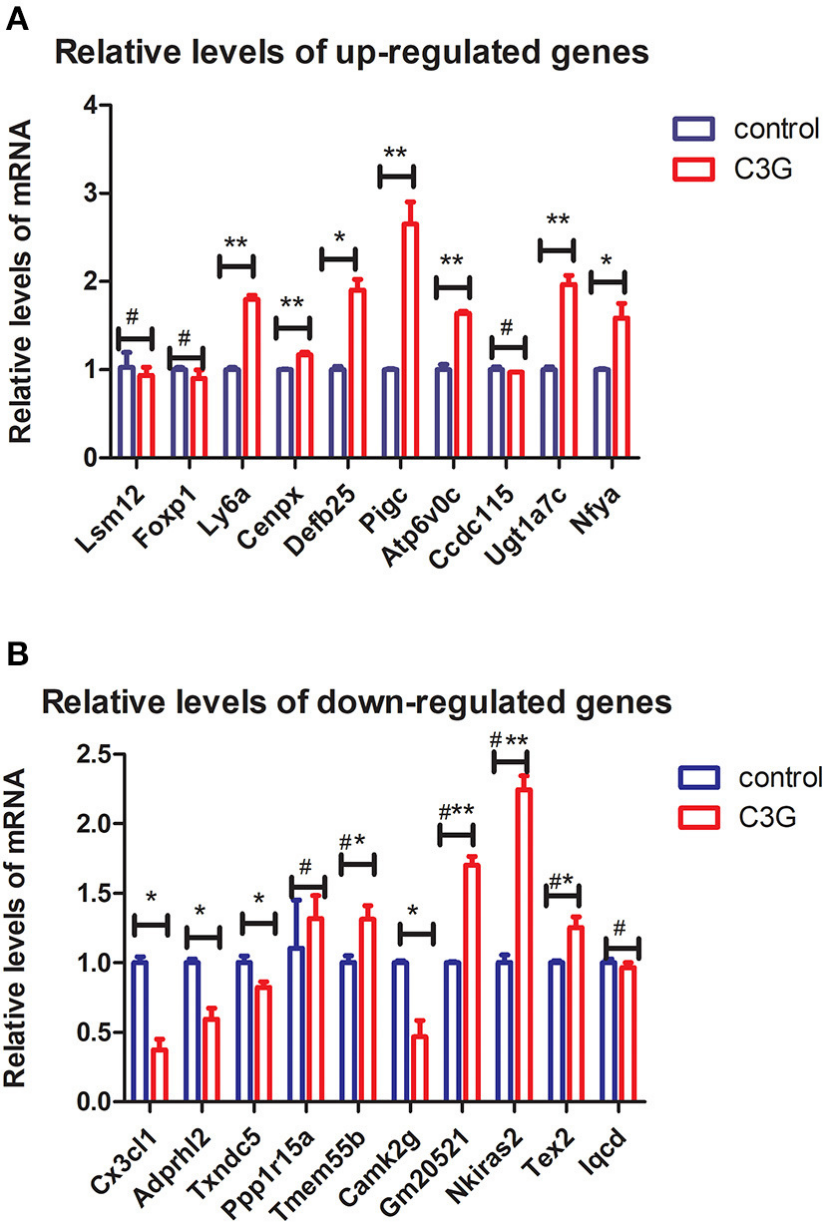
qRT-PCR validation of top DEGs. **(A,B)** Expression levels of top 10 upregulated **(A)** and downregulated **(B)** DEGs evaluated by qRT-PCR (*n* = 3 replicates). *p < 0.05, **p < 0.01.

Among the top 10 upregulated genes identified by RNA-seq, seven [lymphocyte antigen 6 complex, locus A (*Ly6a*); centromere protein X (*Cenpx*), defensin beta 125 (*Defb25*), *Pigc, Atp6v0c*, UDP glucuronosyltransferase family 1 member A7 (*Ugt1a7c*), and *Nfya*] exhibited significant differences in expression in comparative qRT-PCR analysis of C3G-treated and untreated MC3T3-E1 cells, whereas LSM12 homolog (*Lsm12)*, forkhead box P1 (*Foxp1*), and *Ccdc115* mRNA levels did not differ significantly between the groups (Figure 4A). Among the top 10 downregulated DEGs, four [*Cx3cl1*, ADP-ribosylserine hydrolase 12 (*Adprh12*), *Txndc5*, and calcium/calmodulin-dependent protein kinase II gamma (*Camk2g*)] were expressed at significantly lower levels in C3G-treated MC3T3-E1 cells as determined by qRT-PCR, whereas phosphatidylinositol-4,5-bisphosphate 4-phosphatase (*Tmem55b*), *Gm20521, Nkieas2*, and testis expressed 2 (*Tex2*) were upregulated. There were no significant differences in *Ppp1r15a* or IQ motif-containing D [*Iqcd*] expression between treated and untreated cells (Figure 4B).

## Discussion

Osteoblasts are specialized fibroblasts that secrete and mineralize bone matrix and play a critical role in osteoporosis. MC3T3-E1 cells can be induced to differentiate into osteoblasts and are used as *in vitro* models to investigate the molecular mechanisms of osteogenesis (34). In current study, RNA-seq of MC3T3-E1 cells treated with the anthocyanin C3G revealed many DEGs as well as GO terms related to lysosomes—e.g., lysosomal lumen acidification, regulation of lysosomal lumen pH, and lysosome organization. This is consistent with previous report that lysosomes play an important role in biogenesis, mineralization, and trafficking of nanovesicles in osteoblasts (35).

GPI metabolism and lipidation were implicated in the effects of C3G on osteoblasts, as revealed by functional enrichment analyses of DEGs. Phosphorylation of ERK1/2 in osteoblasts promotes the expression of GPI-anchored proteins that maintain cell membrane integrity, which affects osteoblast proliferation (27). Other biological processes including cellular responses to stress (36), cell–to-cell junction organization (19) and cellular responses to DNA damage (37) have been linked to the effects of C3G on osteoblasts. The most significantly enriched KEGG pathways were involved in GPI-anchored protein biosynthesis (upregulated), lupus erythematosus (upregulated), and phagosome (upregulated), and protein processing in endoplasmic reticulum (downregulated). GPI is a ubiquitous glycolipid in eukaryotes (38) that anchors proteins to cell surfaces (39). To date, more than 150 GPI-APs have been identified in mammals (40) including some that are related to bone formation such as ALP, acetylcholinesterase (AChE) (41), and Ly6a (42). The related enzymes PIGC and PIGK play critical roles in GPI synthesis (43). One of the functions of GPI-anchor biosynthesis signaling is to synthesize GPI-Aps. Whether C3G can influence the proliferation and differentiation of osteoblasts by promoting the production of GPI-APs warrants further investigation.

ATP6V0C is a member of the V-ATPase family of enzymes that plays an important role in osteogenesis. Loss of function of V-ATPases results in an osteopetrorickets phenotype due to reduced bone formation (44), and *Atp*6*v*1*h*^+/−^ mice exhibit impaired osteoblast growth as well as abnormal ALP levels (45). V-ATPase deficiency also inhibits osteogenic differentiation and stimulates adipogenic differentiation (46). The CX3CL1/CX3CR1 axis—which has been linked to various diseases including rheumatoid arthritis, spinal cord injury, and osteoarthritis (47)—was identified as a possible target for osteoporosis immunotherapy (48). *Cx3cr1* is expressed in osteoclast precursors, implying that CX3CL1/CX3CR1 signaling can regulate osteoclast differentiation and thus affect the development of osteoporosis (49). *Ly6a* is involved in skull development, fat formation, osteogenesis, and chondrogenesis (50). *Ly6a*-deficient mice exhibit reduced bone formation and osteoclast counts and increased mineralization of trabecular bone, and develop features of consistent with age-related osteoporosis in humans including low bone mass, brittleness, and changes in the mechanical properties of bone (51). Inspired by the results described above, we will perform further studies of investigating the roles of the genes identified in present study,for example via the generation of stable osteoblastic cell lines lacking or overexpressing specific genes. That will be necessary to clarify the molecular mechanisms underlying osteoblast mineralization.

However, the present study had several limitations. Firstly, we did not examine the expression profile of long non-coding RNAs or microRNAs that may be involved in the effects of C3G on osteogenesis. Secondly, while qRT-PCR validation of RNA-seq results is essential, the methods used differ in terms of sensitivity and specificity, which may explain the differences in DEGs that were observed in our two datasets. In order to identify more sensitive targets, we did not use a higher concentration of C3G but instead selected the minimum concentration that was shown to promote MC3T3-E1 cell proliferation in previous experiments. This may be the reason why *Runx2* and other known osteoporosis-related genes were not identified as DEGs in our RNA-seq analysis (34). In future work, immunoblotting and other experimental approaches should be used to validate biological targets of C3G in MC3T3-E1 cells.

## Conclusion

In summary, we identified 51 genes and 5 signaling pathways (GPI-anchor biosynthesis, tuberculosis, systemic lupus erythematous, phagosome, and protein processing in the endoplasmic reticulum) via RNA-seq that may mediate the effects of C3G in osteogenesis. Based on their known involvement in osteoporosis, *Atp6v0c, Cx3cl1*, and *Ly6a* are the most promising targets of C3G action in osteoblasts. These findings provide evidence for the therapeutic potential of C3G in the treatment of osteoporosis and other disorders of bone metabolism.

## Data availability statement

The datasets presented in this study can be found in online repositories. The names of the repository/repositories and accession number(s) can be found in the article/Supplementary material.

## Author contributions

BZ designed the study. LC, BH, YC, and XW performed the experiments. LC and BH analyzed the data. LC and BZ wrote the manuscript. All authors have read and agreed to the publication of this version of the manuscript.

## Funding

This work was supported by the Liaoning Science and Technology Program (Grant No. 20170540879), Shenyang Medical College Masters Science and Technology Innovation Fund Project (Grant No. Y20190508), Shenyang Science and Technology Project (Grant No. 19-112-4-024), and Shenyang Medical College Science and Technology Fund (Grant No. 20182045).

## Conflict of interest

The authors declare that the research was conducted in the absence of any commercial or financial relationships that could be construed as a potential conflict of interest.

## Publisher's note

All claims expressed in this article are solely those of the authors and do not necessarily represent those of their affiliated organizations, or those of the publisher, the editors and the reviewers. Any product that may be evaluated in this article, or claim that may be made by its manufacturer, is not guaranteed or endorsed by the publisher.
