# Supplementary material for: Functional role of cyanidin-3-O-glucoside in osteogenesis: A pilot study based on RNA-seq analysis
Source: Front Nutr. 2022 Sep 30;9:995643. doi: 10.3389/fnut.2022.995643 (PMC9562617; doi:10.3389/fnut.2022.995643)
Supplement: Supplementary file 1 [file Table_1.DOCX]

**Supplementary Table 1. Up-regulated and down-regulated DEGs.**

| **Gene Name** | **Locus** | **log_2_FC** | **Fold Change** | **P value** |
| --- | --- | --- | --- | --- |
| **Up-regulated**  Lsm12  Foxp1  Ly6a  Cenpx  Defb25  Pigc  Atp6v0c  Ccdc115  Ugt1a7c  Nfya  Pigk  Ttll4  Morn5  Atxn7  Wdr38  Lyz2  Moap1  Slc6a9  Lmcd1  Nat8f5  Pfkfb4  Car9  Nit1  Zfp668  Iigp1  Fcgr4  Hjurp  Lyplal1  Tmem53  Serpinb6b  Hist1h2bq  Ccdc17  Glod4  Syngr4  **Down-regulated**  Cx3cl1  Adprhl2  Txndc5  Ppp1r15a  Tmem55b  Camk2g  Gm20521  Nkiras2  Tex2  Iqcd  Faap24  Smad7  Nectin1  Rad1  Fndc4  Ubl4a  Erich1 | chr11:102162497-102185296  chr6:98925338-99522721  chr15:74994877-74998031  chr11:120710942-120713738  chr2:152622356-152623053  chr1:161969186-161973435  chr17:24163866-24169702  chr1:34436670-34439672  chr1:88095062-88220002  chr17:48386885-48409906  chr3:152714100-152980408  chr1:74661745-74703730  chr2:36049458-36079718  chr14:13961440-14107302  chr2:38997476-39006168  chr10:117277331-117282321  chr12:102742230-102743661  chr4:117834506-117875198  chr6:112273758-112330425  chr6:85817218-85820972  chr9:108991778-109032228  chr4:43506966-43513729  chr1:171338008-171345646  chr7:127863047-127876828  chr18:60376029-60392627  chr1:171018920-171029761  chr1:88262471-88277633  chr1:186087731-186117310  chr4:117251951-117268582  chr13:32965209-32979067  chr13:21806612-21810197  chr4:116596654-116600266  chr11:76120987-76243725  chr7:45885222-45896714  chr8:94772009-94782427  chr4:126316047-126321703  chr13:38500079-38528824  chr7:45522916-45526268  chr14:50926068-50930856  chr14:20734875-20794088  chr14:54883441-54898137  chr11:100619244-100627607  chr11:106502147-106613423  chr5:120589016-120607118  chr7:35392152-35396836  chr18:75367529-75395935  chr9:43743984-43807461  chr15:10486018-10499063  chr5:31292242-31296080  chrX:74365718-74373218  chr8:14027561-14090301 | 1.046564283  0.891159799  0.816180742  0.815007475  0.809300798  0.736890497  0.669864627  0.597565105  0.557487141  0.542106983  0.537249944  0.514551598  0.500713936  0.490703963  0.481447842  0.431055633  0.421601287  0.419716909  0.409598611  0.400537818  0.390809129  0.368468224  0.364365943  0.350212178  0.31955274  0.317002676  0.30618316  0.296178846  0.295875239  0.287631376  0.275532534  0.275086427  0.271983327  0.266333262  -2.091787797  -1.202617707  -1.139194392  -0.768270084  -0.611329124  -0.538663727  -0.483788268  -0.444943707  -0.387598065  -0.375232269  -0.372403732  -0.315484925  -0.305339274  -0.296312356  -0.295740336  -0.28087341  -0.27159424 | 2.065604835  1.854666512  1.760738601  1.759307267  1.752361954  1.666579913  1.590923679  1.513160587  1.471703598  1.456097522  1.451203604  1.428550065  1.414913577  1.405130342  1.396144088  1.348219721  1.339413382  1.337665048  1.328316196  1.319999898  1.311128538  1.290981408  1.287315737  1.274748092  1.247943605  1.245739726  1.236432225  1.227887891  1.227629516  1.220634587  1.21044081  1.210066578  1.207466637  1.202747051  0.234589802  0.43448621  0.45401303  0.587121061  0.654593361  0.688408241  0.715097434  0.73461298  0.764401193  0.770981277  0.77249434  0.803580842  0.809251885  0.814331234  0.814654175  0.823092565  0.828403618 | 0.025653872  0.003332163  0.012549902  0.025588789  0.020646807  0.003772964  0.044304926  0.006804133  0.049272618  0.016586163  0.00836339  0.024546391  0.012038288  0.030420151  0.0012692  0.018717792  0.026998184  0.025600133  0.047423897  9.00556E-05  0.031845814  0.024465123  0.033733977  0.04240304  0.033322892  0.042672745  0.049600498  0.023204719  0.04815002  0.010728991  0.047410707  0.014402721  0.046104345  0.000460067  0.000885861  0.022127739  0.03408987  0.022259685  0.048048487  0.005173393  0.026555378  0.014396726  0.00213289  0.041334286  0.040002938  0.019327037  0.026706926  0.006486885  0.036161146  0.005759681  0.026233272 |

DEG, differentially expressed gene.
