# Supplementary material for: Functional role of cyanidin-3-O-glucoside in osteogenesis: A pilot study based on RNA-seq analysis
Source: Front Nutr. 2022 Sep 30;9:995643. doi: 10.3389/fnut.2022.995643 (PMC9562617; doi:10.3389/fnut.2022.995643)
Supplement: Supplementary file 2 [file Table_2.DOCX]

**Supplementary Table 2.** **The enriched BP terms of up-regulated and down-regulated DEGs.**

| **ID** | **Term** | **DEG(s)** | **p-value** | **FDR** | **Enrichment** |
| --- | --- | --- | --- | --- | --- |
| Up-regulated |  |  |  |  |  |
| GO:0007042 | lysosomal_lumen_acidification | Atp6v0c//Ccdc115 | 0.000051714 | 0.031235009 | 4.286395308 |
| GO:0035751 | regulation_of_lysosomal_lumen_pH | Atp6v0c//Ccdc115 | 0.000121475 | 0.036685321 | 3.915514623 |
| GO:0007035 | vacuolar_acidification | Atp6v0c//Ccdc115 | 0.000280141 | 0.056401623 | 3.552624083 |
| GO:0042157 | lipoprotein_metabolic_process | Lyplal1//Pigc//Pigk | 0.000496347 | 0.060730184 | 3.30421487 |
| GO:0034508 | centromere_complex_assembly | Cenpx//Hjurp | 0.000502733 | 0.060730184 | 3.298662336 |
| GO:0051452 | intracellular_pH_reduction | Atp6v0c//Ccdc115 | 0.000686147 | 0.064043499 | 3.163582969 |
| GO:0006506 | GPI_anchor_biosynthetic_process | Pigc//Pigk | 0.000841882 | 0.064043499 | 3.074749022 |
| GO:0045851 | pH_reduction | Atp6v0c//Ccdc115 | 0.00089723 | 0.064043499 | 3.047095975 |
| GO:0006505 | GPI_anchor_metabolic_process | Pigc//Pigk | 0.000954291 | 0.064043499 | 3.020319376 |
| GO:0009247 | glycolipid_biosynthetic_process | Pigc//Pigk | 0.002633397 | 0.083516259 | 2.579483631 |
| GO:0006661 | phosphatidylinositol_biosynthetic_process | Pigc//Pigk | 0.002728574 | 0.083516259 | 2.564064333 |
| GO:0007040 | lysosome_organization | Atp6v0c//Ccdc115 | 0.003125278 | 0.089888944 | 2.505111364 |
| GO:0010611 | regulation_of_cardiac_muscle_hypertrophy | Foxp1//Lmcd1 | 0.003333169 | 0.09151063 | 2.477142712 |
| GO:0051453 | regulation_of_intracellular_pH | Atp6v0c//Ccdc115 | 0.00471189 | 0.118582575 | 2.326804819 |
| GO:0030641 | regulation_of_cellular_pH | Atp6v0c//Ccdc115 | 0.005351685 | 0.123561881 | 2.271509421 |
| GO:0006664 | glycolipid_metabolic_process | Pigc//Pigk | 0.005753672 | 0.124114921 | 2.240054914 |
| GO:0006497 | protein_lipidation | Pigc//Pigk | 0.006453472 | 0.124938711 | 2.190206598 |
| GO:0042158 | lipoprotein_biosynthetic_process | Pigc//Pigk | 0.007190104 | 0.124938711 | 2.143264848 |
| GO:0006885 | regulation_of_pH | Atp6v0c//Ccdc115 | 0.007494955 | 0.124938711 | 2.125230961 |
| GO:0046474 | glycerophospholipid_biosynthetic_process | Pigc//Pigk | 0.008121963 | 0.124938711 | 2.090338983 |
| GO:0050829 | defense_response_to_Gram-negative_bacterium | Iigp1//Lyz2 | 0.009615919 | 0.129479232 | 2.017009201 |
| GO:0046488 | phosphatidylinositol_metabolic_process | Pigc//Pigk | 0.012355161 | 0.136022963 | 1.908151603 |
| GO:0007033 | vacuole_organization | Atp6v0c//Ccdc115 | 0.012743365 | 0.137446292 | 1.894715885 |
| GO:0008654 | phospholipid_biosynthetic_process | Pigc//Pigk | 0.017134186 | 0.159216131 | 1.766136515 |
| GO:0005996 | monosaccharide_metabolic_process | Pfkfb4//Ugt1a7c | 0.018491331 | 0.164246524 | 1.733031839 |
| GO:0006643 | membrane_lipid_metabolic_process | Pigc//Pigk | 0.021093558 | 0.169346728 | 1.675850168 |
| GO:0065004 | protein-DNA_complex_assembly | Cenpx//Hjurp | 0.022824617 | 0.169346728 | 1.641596494 |
| GO:0009617 | response_to_bacterium | Fcgr4//Iigp1//Ly6a//Lyz2 | 0.023669645 | 0.170196021 | 1.62580825 |
| GO:0006310 | DNA_recombination | Cenpx//Foxp1 | 0.027534659 | 0.184788155 | 1.560120301 |
| GO:0046903 | secretion | Car9//Foxp1//Slc6a9 | 0.03138244 | 0.195412309 | 1.503313288 |
| GO:0006650 | glycerophospholipid_metabolic_process | Pigc//Pigk | 0.032310935 | 0.195552435 | 1.490650469 |
| GO:0034645 | cellular_macromolecule_biosynthetic_process | Atxn7//Cenpx//Foxp1//Lmcd1//Nfya//Pigc//Pigk//Zfp668 | 0.034704301 | 0.196037871 | 1.4596167 |
| GO:0090257 | regulation_of_muscle_system_process | Foxp1//Lmcd1 | 0.037389147 | 0.198113087 | 1.427254441 |
| GO:0007059 | chromosome_segregation | Cenpx//Hjurp | 0.048050263 | 0.211438028 | 1.318304231 |
| GO:0051707 | response_to_other_organism | Fcgr4//Iigp1//Ly6a//Lyz2 | 0.050449673 | 0.213664384 | 1.297141642 |
| GO:0043207 | response_to_external_biotic_stimulus | Fcgr4//Iigp1//Ly6a//Lyz2 | 0.050750712 | 0.213664384 | 1.294557859 |
| GO:0009607 | response_to_biotic_stimulus | Fcgr4//Iigp1//Ly6a//Lyz2 | 0.055387572 | 0.224142983 | 1.256587675 |
| GO:0046486 | glycerolipid_metabolic_process | Pigc//Pigk | 0.056777941 | 0.224142983 | 1.245820361 |
| GO:0006644 | phospholipid_metabolic_process | Pigc//Pigk | 0.059680899 | 0.231125451 | 1.224164645 |
| GO:0019637 | organophosphate_metabolic_process | Pfkfb4//Pigc//Pigk | 0.09994725 | 0.308261599 | 1.000229152 |
| Down-regulated | | | | | |
| GO:0033554 | cellular_response_to_stress | Adprhl2//Faap24//Gm20521//Ppp1r15a//Rad1 | 0.001529696 | 0.114686788 | 2.815395 |
| GO:0006950 | response_to_stress | Adprhl2//Camk2g//Cx3cl1//Faap24//Gm20521//Ppp1r15a//Rad1 | 0.002156548 | 0.114686788 | 2.66624084 |
| GO:0033135 | regulation_of_peptidyl-serine_phosphorylation | Ppp1r15a//Smad7 | 0.004392513 | 0.114686788 | 2.357286896 |
| GO:0045216 | cell-cell_junction_organization | Nectin1//Smad7 | 0.004863323 | 0.114686788 | 2.313066923 |
| GO:0051179 | localization | Camk2g//Cx3cl1//Nectin1//Ppp1r15a//Smad7//Tex2//Txndc5//Ubl4a | 0.006595983 | 0.114686788 | 2.180720442 |
| GO:0006974 | cellular_response_to_DNA_damage_stimulus | Faap24//Gm20521//Rad1 | 0.008369015 | 0.114686788 | 2.077325642 |
| GO:0090257 | regulation_of_muscle_system_process | Camk2g//Smad7 | 0.009416313 | 0.114686788 | 2.026119096 |
| GO:2001234 | negative_regulation_of_apoptotic_signaling_pathway | Cx3cl1//Gm20521 | 0.009662976 | 0.114686788 | 2.014889099 |
| GO:0022409 | positive_regulation_of_cell-cell_adhesion | Cx3cl1//Smad7 | 0.009996352 | 0.114686788 | 2.000158469 |
| GO:0001818 | negative_regulation_of_cytokine_production | Cx3cl1//Smad7 | 0.010420254 | 0.114686788 | 1.982121684 |
| GO:0033036 | macromolecule_localization | Nectin1//Ppp1r15a//Smad7//Tex2//Ubl4a | 0.010519586 | 0.114686788 | 1.978001353 |
| GO:1903522 | regulation_of_blood_circulation | Cx3cl1//Smad7 | 0.011649223 | 0.114686788 | 1.933703051 |
| GO:0006979 | response_to_oxidative_stress | Adprhl2//Camk2g | 0.020382567 | 0.143648566 | 1.690741126 |
| GO:0022407 | regulation_of_cell-cell_adhesion | Cx3cl1//Smad7 | 0.024701722 | 0.153786446 | 1.607272771 |
| GO:0045785 | positive_regulation_of_cell_adhesion | Cx3cl1//Smad7 | 0.027381072 | 0.1598724 | 1.562549551 |
| GO:2001233 | regulation_of_apoptotic_signaling_pathway | Cx3cl1//Gm20521 | 0.029498926 | 0.161009167 | 1.53019379 |
| GO:0008104 | protein_localization | Nectin1//Ppp1r15a//Smad7//Ubl4a | 0.029549536 | 0.161009167 | 1.529449327 |
| GO:0001933 | negative_regulation_of_protein_phosphorylation | Ppp1r15a//Smad7 | 0.029768223 | 0.161009167 | 1.526247083 |
| GO:0006281 | DNA_repair | Faap24//Rad1 | 0.030174053 | 0.161009167 | 1.520366355 |
| GO:0048514 | blood_vessel_morphogenesis | Cx3cl1//Smad7 | 0.030582134 | 0.161009167 | 1.514532214 |
| GO:0042391 | regulation_of_membrane_potential | Cx3cl1//Smad7 | 0.034069126 | 0.161009167 | 1.467639009 |
| GO:0050896 | response_to_stimulus | Adprhl2//Camk2g//Cx3cl1//Faap24//Gm20521//Nectin1//Nkiras2//Ppp1r15a//Rad1//Smad7 | 0.034111964 | 0.161009167 | 1.467093279 |
| GO:0042326 | negative_regulation_of_phosphorylation | Ppp1r15a//Smad7 | 0.035361523 | 0.161009167 | 1.451469042 |
| GO:0006935 | chemotaxis | Cx3cl1//Nectin1 | 0.038003769 | 0.165454643 | 1.42017333 |
| GO:0009968 | negative_regulation_of_signal_transduction | Cx3cl1//Gm20521//Smad7 | 0.041581527 | 0.166326109 | 1.381099565 |
| GO:0031401 | positive_regulation_of_protein_modification_process | Cx3cl1//Ppp1r15a//Smad7 | 0.042655727 | 0.168347935 | 1.370022654 |
| GO:0001568 | blood_vessel_development | Cx3cl1//Smad7 | 0.045890859 | 0.170517796 | 1.338273817 |
| GO:0033365 | protein_localization_to_organelle | Ppp1r15a//Ubl4a | 0.051294575 | 0.175555453 | 1.289928561 |
| GO:0001944 | vasculature_development | Cx3cl1//Smad7 | 0.051797024 | 0.175555453 | 1.285695192 |
| GO:0034613 | cellular_protein_localization | Ppp1r15a//Smad7//Ubl4a | 0.052363318 | 0.175555453 | 1.280972845 |
| GO:0023057 | negative_regulation_of_signaling | Cx3cl1//Gm20521//Smad7 | 0.054080565 | 0.175555453 | 1.266958779 |
| GO:0072358 | cardiovascular_system_development | Cx3cl1//Smad7 | 0.054165853 | 0.175555453 | 1.266274414 |
| GO:0030001 | metal_ion_transport | Camk2g//Nectin1 | 0.059195895 | 0.178008399 | 1.227708412 |
| GO:0001932 | regulation_of_protein_phosphorylation | Cx3cl1//Ppp1r15a//Smad7 | 0.064188504 | 0.179008021 | 1.192542749 |
| GO:0001817 | regulation_of_cytokine_production | Cx3cl1//Smad7 | 0.066211533 | 0.181309615 | 1.179066357 |
| GO:0030155 | regulation_of_cell_adhesion | Cx3cl1//Smad7 | 0.067684839 | 0.181309615 | 1.169508598 |
| GO:0051128 | regulation_of_cellular_component_organization | Cx3cl1//Nectin1//Ppp1r15a//Rad1 | 0.071130439 | 0.187152088 | 1.147944511 |
| GO:0051345 | positive_regulation_of_hydrolase_activity | Cx3cl1//Ppp1r15a | 0.071794283 | 0.188062902 | 1.143910135 |
| GO:0006259 | DNA_metabolic_process | Faap24//Rad1 | 0.073119274 | 0.188395545 | 1.13596813 |
| GO:0032270 | positive_regulation_of_cellular_protein_metabolic_process | Cx3cl1//Ppp1r15a//Smad7 | 0.077311295 | 0.192846233 | 1.111757053 |
| GO:0042325 | regulation_of_phosphorylation | Cx3cl1//Ppp1r15a//Smad7 | 0.07755401 | 0.192846233 | 1.11039574 |
| GO:0035239 | tube_morphogenesis | Cx3cl1//Smad7 | 0.079280064 | 0.193413456 | 1.10083601 |
| GO:0051234 | establishment_of_localization | Camk2g//Nectin1//Tex2//Txndc5//Ubl4a | 0.0818442 | 0.193949627 | 1.08701209 |
| GO:0007155 | cell_adhesion | Cx3cl1//Nectin1 | 0.090042746 | 0.203964294 | 1.04555127 |
| GO:0051247 | positive_regulation_of_protein_metabolic_process | Cx3cl1//Ppp1r15a//Smad7 | 0.090267981 | 0.203964294 | 1.044466269 |
| GO:0043086 | negative_regulation_of_catalytic_activity | Ppp1r15a//Smad7 | 0.09558576 | 0.208039596 | 1.019606801 |

BP: biological process; DEG: differentially expressed gene; FDR: false discovery rate.
