# Supplementary material for: Functional role of cyanidin-3-O-glucoside in osteogenesis: A pilot study based on RNA-seq analysis
Source: Front Nutr. 2022 Sep 30;9:995643. doi: 10.3389/fnut.2022.995643 (PMC9562617; doi:10.3389/fnut.2022.995643)
Supplement: Supplementary file 3 [file Table_3.DOCX]

**S**upplementary Table 3. The enriched CC terms of up-regulated and down-regulated DEGs.

| **ID** | **Term** | **DEG(s)** | **p-value** | **FDR** | **Enrichment** |
| --- | --- | --- | --- | --- | --- |
| Up-regulated |  |  |  |  |  |
| GO:0016471 | vacuolar_proton-transporting_V-type_ATPase_complex | Atp6v0c//Ccdc115 | 0.026702739 | 3.564939487 | 81.35640138 |
| GO:0033176 | proton-transporting_V-type_ATPase_complex | Atp6v0c//Ccdc115 | 0.026702739 | 3.297720067 | 60.13299233 |
| GO:0005789 | endoplasmic_reticulum_membrane | Ccdc115//Iigp1//Pigc//Pigk | 0.101831023 | 2.31630488 | 5.786857002 |
| GO:0042175 | nuclear_outer_membrane-endoplasmic_reticulum_membrane_network | Ccdc115//Iigp1//Pigc//Pigk | 0.101831023 | 2.244563369 | 5.521192908 |
| GO:0005798 | Golgi-associated_vesicle | Ccdc115//Lyz2 | 0.104645797 | 1.832003238 | 10.80514706 |
| GO:0000776 | kinetochore | Cenpx//Hjurp | 0.104645797 | 1.819264023 | 10.63891403 |
| GO:0005774 | vacuolar_membrane | Atp6v0c//Ccdc115 | 0.122012421 | 1.66014822 | 8.753536858 |
| GO:0030135 | coated_vesicle | Ccdc115//Lyz2 | 0.145385334 | 1.559595155 | 7.726585606 |
| GO:0005783 | endoplasmic_reticulum | Ccdc115//Iigp1//Lyz2//Nat8f5//Pigc//Pigk | 0.145385334 | 1.540196278 | 2.522295727 |
| GO:0000775 | chromosome,_centromeric_region | Cenpx//Hjurp | 0.145385334 | 1.520362585 | 7.35669587 |
| GO:0005622 | intracellular | Atp6v0c//Atxn7//Ccdc115//Cenpx//Foxp1//Glod4//Hist1h2bq//Hjurp//Iigp1//Lmcd1//Ly6a//Lyplal1//Lyz2//Moap1//Nat8f5//Nfya//Nit1//Pfkfb4//Pigc//Pigk//Serpinb6b//Syngr4//Ttll4//Ugt1a7c//Zfp668 | 0.165510168 | 1.369880914 | 1.274004075 |
| GO:0005623 | cell | Atp6v0c//Atxn7//Car9//Ccdc115//Cenpx//Fcgr4//Foxp1//Glod4//Hist1h2bq//Hjurp//Iigp1//Lmcd1//Ly6a//Lyplal1//Lyz2//Moap1//Nat8f5//Nfya//Nit1//Pfkfb4//Pigc//Pigk//Serpinb6b//Slc6a9//Syngr4//Ttll4//Ugt1a7c//Zfp668 | 0.165510168 | 1.344138062 | 1.215952244 |
| GO:0030133 | transport_vesicle | Lyz2//Syngr4 | 0.222497125 | 1.185084761 | 4.78567067 |
| GO:0005737 | cytoplasm | Atp6v0c//Atxn7//Ccdc115//Foxp1//Glod4//Hist1h2bq//Hjurp//Iigp1//Lmcd1//Lyplal1//Lyz2//Moap1//Nat8f5//Nit1//Pfkfb4//Pigc//Pigk//Serpinb6b//Syngr4//Ttll4 | 0.262971095 | 1.061329807 | 1.286446678 |
| GO:0043231 | intracellular_membrane-bounded_organelle | Atp6v0c//Atxn7//Ccdc115//Cenpx//Foxp1//Glod4//Hist1h2bq//Hjurp//Iigp1//Lmcd1//Lyz2//Moap1//Nat8f5//Nfya//Nit1//Pigc//Pigk//Ugt1a7c//Zfp668 | 0.269432415 | 1.027586144 | 1.293596419 |
| GO:0043227 | membrane-bounded_organelle | Atp6v0c//Atxn7//Ccdc115//Cenpx//Foxp1//Glod4//Hist1h2bq//Hjurp//Iigp1//Lmcd1//Lyz2//Moap1//Nat8f5//Nfya//Nit1//Pigc//Pigk//Syngr4//Ugt1a7c//Zfp668 | 0.269432415 | 1.015072424 | 1.272480287 |
| GO:0016471 | vacuolar_proton-transporting_V-type_ATPase_complex | Atp6v0c//Ccdc115 | 0.026702739 | 3.564939487 | 81.35640138 |
| GO:0033176 | proton-transporting_V-type_ATPase_complex | Atp6v0c//Ccdc115 | 0.026702739 | 3.297720067 | 60.13299233 |
| GO:0005789 | endoplasmic_reticulum_membrane | Ccdc115//Iigp1//Pigc//Pigk | 0.101831023 | 2.31630488 | 5.786857002 |
| GO:0042175 | nuclear_outer_membrane-endoplasmic_reticulum_membrane_network | Ccdc115//Iigp1//Pigc//Pigk | 0.101831023 | 2.244563369 | 5.521192908 |
| GO:0005798 | Golgi-associated_vesicle | Ccdc115//Lyz2 | 0.104645797 | 1.832003238 | 10.80514706 |
| GO:0000776 | kinetochore | Cenpx//Hjurp | 0.104645797 | 1.819264023 | 10.63891403 |
| GO:0005774 | vacuolar_membrane | Atp6v0c//Ccdc115 | 0.122012421 | 1.66014822 | 8.753536858 |
| GO:0030135 | coated_vesicle | Ccdc115//Lyz2 | 0.145385334 | 1.559595155 | 7.726585606 |
| GO:0005783 | endoplasmic_reticulum | Ccdc115//Iigp1//Lyz2//Nat8f5//Pigc//Pigk | 0.145385334 | 1.540196278 | 2.522295727 |
| GO:0000775 | chromosome,_centromeric_region | Cenpx//Hjurp | 0.145385334 | 1.520362585 | 7.35669587 |
| GO:0005622 | intracellular | Atp6v0c//Atxn7//Ccdc115//Cenpx//Foxp1//Glod4//Hist1h2bq//Hjurp//Iigp1//Lmcd1//Ly6a//Lyplal1//Lyz2//Moap1//Nat8f5//Nfya//Nit1//Pfkfb4//Pigc//Pigk//Serpinb6b//Syngr4//Ttll4//Ugt1a7c//Zfp668 | 0.165510168 | 1.369880914 | 1.274004075 |
| GO:0005623 | cell | Atp6v0c//Atxn7//Car9//Ccdc115//Cenpx//Fcgr4//Foxp1//Glod4//Hist1h2bq//Hjurp//Iigp1//Lmcd1//Ly6a//Lyplal1//Lyz2//Moap1//Nat8f5//Nfya//Nit1//Pfkfb4//Pigc//Pigk//Serpinb6b//Slc6a9//Syngr4//Ttll4//Ugt1a7c//Zfp668 | 0.165510168 | 1.344138062 | 1.215952244 |
| GO:0030133 | transport_vesicle | Lyz2//Syngr4 | 0.222497125 | 1.185084761 | 4.78567067 |
| GO:0005737 | cytoplasm | Atp6v0c//Atxn7//Ccdc115//Foxp1//Glod4//Hist1h2bq//Hjurp//Iigp1//Lmcd1//Lyplal1//Lyz2//Moap1//Nat8f5//Nit1//Pfkfb4//Pigc//Pigk//Serpinb6b//Syngr4//Ttll4 | 0.262971095 | 1.061329807 | 1.286446678 |
| GO:0043231 | intracellular_membrane-bounded_organelle | Atp6v0c//Atxn7//Ccdc115//Cenpx//Foxp1//Glod4//Hist1h2bq//Hjurp//Iigp1//Lmcd1//Lyz2//Moap1//Nat8f5//Nfya//Nit1//Pigc//Pigk//Ugt1a7c//Zfp668 | 0.269432415 | 1.027586144 | 1.293596419 |
| GO:0043227 | membrane-bounded_organelle | Atp6v0c//Atxn7//Ccdc115//Cenpx//Foxp1//Glod4//Hist1h2bq//Hjurp//Iigp1//Lmcd1//Lyz2//Moap1//Nat8f5//Nfya//Nit1//Pigc//Pigk//Syngr4//Ugt1a7c//Zfp668 | 0.269432415 | 1.015072424 | 1.272480287 |
| Down-regulated | | | | | |
| GO:0016342 | catenin_complex | Nectin1//Smad7 | 0.000162428 | 0.012506923 | 3.789340246 |
| GO:0005622 | intracellular | Adprhl2//Camk2g//Cx3cl1//Faap24//Fndc4//Gm20521//Iqcd//Nectin1//Nkiras2//Ppp1r15a//Rad1//Smad7//Tex2//Txndc5//Ubl4a | 0.001923219 | 0.041375533 | 2.715971321 |
| GO:0005913 | cell-cell_adherens_junction | Nectin1//Smad7 | 0.002149378 | 0.041375533 | 2.66768713 |
| GO:0005783 | endoplasmic_reticulum | Camk2g//Fndc4//Ppp1r15a//Tex2//Txndc5 | 0.003790593 | 0.047879261 | 2.421292814 |
| GO:0005741 | mitochondrial_outer_membrane | Gm20521//Ppp1r15a | 0.005968628 | 0.047879261 | 2.224125515 |
| GO:0019897 | extrinsic_component_of_plasma_membrane | Nectin1//Smad7 | 0.006103817 | 0.047879261 | 2.214398524 |
| GO:0019867 | outer_membrane | Gm20521//Ppp1r15a | 0.007235551 | 0.047879261 | 2.140528406 |
| GO:0031968 | organelle_outer_membrane | Gm20521//Ppp1r15a | 0.007235551 | 0.047879261 | 2.140528406 |
| GO:0043231 | intracellular_membrane-bounded_organelle | Adprhl2//Camk2g//Faap24//Fndc4//Gm20521//Nectin1//Ppp1r15a//Rad1//Smad7//Tex2//Txndc5//Ubl4a | 0.010190816 | 0.056049487 | 1.991791047 |
| GO:0043229 | intracellular_organelle | Adprhl2//Camk2g//Faap24//Fndc4//Gm20521//Iqcd//Nectin1//Ppp1r15a//Rad1//Smad7//Tex2//Txndc5//Ubl4a | 0.011342463 | 0.058224644 | 1.945292627 |
| GO:0005912 | adherens_junction | Nectin1//Smad7 | 0.014478929 | 0.069679844 | 1.839263571 |
| GO:0019898 | extrinsic_component_of_membrane | Nectin1//Smad7 | 0.01644454 | 0.070004026 | 1.783978267 |
| GO:0005623 | cell | Adprhl2//Camk2g//Cx3cl1//Faap24//Fndc4//Gm20521//Iqcd//Nectin1//Nkiras2//Ppp1r15a//Rad1//Smad7//Tex2//Txndc5//Ubl4a | 0.016874316 | 0.070004026 | 1.772773829 |
| GO:0005737 | cytoplasm | Adprhl2//Camk2g//Cx3cl1//Fndc4//Gm20521//Iqcd//Nkiras2//Ppp1r15a//Smad7//Tex2//Txndc5//Ubl4a | 0.017273721 | 0.070004026 | 1.762614105 |
| GO:0043227 | membrane-bounded_organelle | Adprhl2//Camk2g//Faap24//Fndc4//Gm20521//Nectin1//Ppp1r15a//Rad1//Smad7//Tex2//Txndc5//Ubl4a | 0.019083723 | 0.073472334 | 1.719336892 |
| GO:0005654 | nucleoplasm | Adprhl2//Faap24//Rad1//Smad7//Ubl4a | 0.02601856 | 0.095401386 | 1.584716745 |
| GO:0005911 | cell-cell_junction | Nectin1//Smad7 | 0.037639706 | 0.120760724 | 1.424353774 |
| GO:0098797 | plasma_membrane_protein_complex | Nectin1//Smad7 | 0.050243143 | 0.143286001 | 1.2989232 |
| GO:0016020 | membrane | Camk2g//Cx3cl1//Fndc4//Gm20521//Nectin1//Nkiras2//Ppp1r15a//Smad7//Tex2//Ubl4a | 0.054328866 | 0.144252506 | 1.26496936 |
| GO:0031966 | mitochondrial_membrane | Gm20521//Ppp1r15a | 0.05678755 | 0.145754711 | 1.24574687 |
| GO:0005740 | mitochondrial_envelope | Gm20521//Ppp1r15a | 0.063988266 | 0.158938597 | 1.193899657 |
| GO:0042995 | cell_projection | Camk2g//Cx3cl1//Iqcd//Nectin1 | 0.070438946 | 0.169493715 | 1.152187149 |
| GO:0031981 | nuclear_lumen | Adprhl2//Faap24//Rad1//Smad7//Ubl4a | 0.077200511 | 0.180134525 | 1.112379827 |
| GO:0012505 | endomembrane_system | Camk2g//Fndc4//Ppp1r15a//Tex2//Txndc5 | 0.092951583 | 0.198813109 | 1.031743208 |

CC: cellular component; DEG: differentially expressed gene; FDR: false discovery rate.
