# Supplementary material for: Functional role of cyanidin-3-O-glucoside in osteogenesis: A pilot study based on RNA-seq analysis
Source: Front Nutr. 2022 Sep 30;9:995643. doi: 10.3389/fnut.2022.995643 (PMC9562617; doi:10.3389/fnut.2022.995643)
Supplement: Supplementary file 4 [file Table_4.DOCX]

**Supplementary Table 4.** **The enriched MF terms of up-regulated and down-regulated DEGs.**

| **ID** | **Term** | **DEG(s)** | **p-value** | **FDR** | **Enrichment** |
| --- | --- | --- | --- | --- | --- |
| Up-regulated |  |  |  |  |  |
| GO:0044389 | vacuolar_proton-transporting_V-type_ATPase_complex | Atp6v0c//Hist1h2bq//Moap1 | 0.010400404 | 0.13248435 | 1.982949791 |
| GO:0008194 | proton-transporting_V-type_ATPase_complex | Pigc//Ugt1a7c | 0.017711952 | 0.151017694 | 1.751733579 |
| GO:0016758 | endoplasmic_reticulum_membrane | Pigc//Ugt1a7c | 0.030734777 | 0.187228024 | 1.512369933 |
| GO:0000978 | nuclear_outer_membrane-endoplasmic_reticulum_membrane_network | Foxp1//Nfya//Zfp668 | 0.031204671 | 0.187228024 | 1.505780397 |
| GO:0000987 | Golgi-associated_vesicle | Foxp1//Nfya//Zfp668 | 0.032881619 | 0.189750546 | 1.483046807 |
| GO:0003690 | kinetochore | Cenpx//Foxp1//Nfya//Zfp668 | 0.042913177 | 0.210664686 | 1.367409335 |
| GO:0016757 | vacuolar_membrane | Pigc//Ugt1a7c | 0.055498485 | 0.264433956 | 1.255718875 |
| GO:0016787 | coated_vesicle | Atp6v0c//Iigp1//Lyplal1//Lyz2//Nit1//Pfkfb4//Pigk | 0.066120052 | 0.281488233 | 1.179666816 |
| GO:0031625 | endoplasmic_reticulum | Atp6v0c//Moap1 | 0.069000484 | 0.281488233 | 1.16114786 |
| GO:0015077 | chromosome,_centromeric_region | Atp6v0c//Slc6a9 | 0.08862934 | 0.302369172 | 1.052422484 |
| GO:0000977 | intracellular | Foxp1//Nfya//Zfp668 | 0.089590866 | 0.302369172 | 1.047736267 |
| GO:0001012 | cell | Foxp1//Nfya//Zfp668 | 0.091825805 | 0.303587357 | 1.037035254 |
| GO:0044389 | transport_vesicle | Atp6v0c//Hist1h2bq//Moap1 | 0.010400404 | 0.13248435 | 1.982949791 |
| GO:0008194 | cytoplasm | Pigc//Ugt1a7c | 0.017711952 | 0.151017694 | 1.751733579 |
| GO:0016758 | intracellular_membrane-bounded_organelle | Pigc//Ugt1a7c | 0.030734777 | 0.187228024 | 1.512369933 |
| GO:0000978 | membrane-bounded_organelle | Foxp1//Nfya//Zfp668 | 0.031204671 | 0.187228024 | 1.505780397 |
| GO:0000987 | vacuolar_proton-transporting_V-type_ATPase_complex | Foxp1//Nfya//Zfp668 | 0.032881619 | 0.189750546 | 1.483046807 |
| GO:0003690 | proton-transporting_V-type_ATPase_complex | Cenpx//Foxp1//Nfya//Zfp668 | 0.042913177 | 0.210664686 | 1.367409335 |
| GO:0016757 | endoplasmic_reticulum_membrane | Pigc//Ugt1a7c | 0.055498485 | 0.264433956 | 1.255718875 |
| GO:0016787 | nuclear_outer_membrane-endoplasmic_reticulum_membrane_network | Atp6v0c//Iigp1//Lyplal1//Lyz2//Nit1//Pfkfb4//Pigk | 0.066120052 | 0.281488233 | 1.179666816 |
| GO:0031625 | Golgi-associated_vesicle | Atp6v0c//Moap1 | 0.069000484 | 0.281488233 | 1.16114786 |
| GO:0015077 | kinetochore | Atp6v0c//Slc6a9 | 0.08862934 | 0.302369172 | 1.052422484 |
| GO:0000977 | vacuolar_membrane | Foxp1//Nfya//Zfp668 | 0.089590866 | 0.302369172 | 1.047736267 |
| GO:0001012 | coated_vesicle | Foxp1//Nfya//Zfp668 | 0.091825805 | 0.303587357 | 1.037035254 |
| Down-regulated | | | | | |
| GO:0050839 | cell_adhesion_molecule_binding | Cx3cl1//Nectin1 | 0.011217627 | 0.109932742 | 1.950099014 |
| GO:0005126 | cytokine_receptor_binding | Cx3cl1//Smad7 | 0.020811246 | 0.156884775 | 1.681701925 |
| GO:0042803 | protein_homodimerization_activity | Camk2g//Gm20521//Nectin1 | 0.023253865 | 0.162777058 | 1.633504845 |
| GO:0046982 | protein_heterodimerization_activity | Gm20521//Nectin1 | 0.059294987 | 0.215218842 | 1.226982022 |
| GO:0046983 | protein_dimerization_activity | Camk2g//Gm20521//Nectin1 | 0.066537251 | 0.21735502 | 1.176935145 |

MF: molecular function; DEG: differentially expressed gene; FDR: false discovery rate.
